# Understanding the Interaction Between Infant Feeding and Maternal Mental Health and Well-Being in Women who Experience Psychological Birth Trauma: A Scoping Review Protocol

**DOI:** 10.12688/hrbopenres.14414.1

**Published:** 2026-04-21

**Authors:** Roisin O'Donovan, Zhuming Bao, Elaine Lehane, Aoife Long, Karen Matvienko-Sikar, Helen Mulcahy, Margaret Murphy, Orla Sheehan, Siobhan Ward, Patricia Leahy-Warren

**Affiliations:** 1 University College Cork School of Nursing and Midwifery, Cork, County Cork, Ireland; 2 University College Cork School of Public Health, Cork, County Cork, Ireland; 3 La Leche League Ireland, Cork, Ireland

**Keywords:** Traumatic Birth; Infant feeding; Maternal Mental Health; Well-Being; Post-Traumatic Growth

## Abstract

**Objective:**

To review the literature to understand women’s experiences of and the interactions between infant feeding, mental health and wellbeing following psychological birth trauma.

**Introduction:**

Childbirth can be a traumatic experience for some women and can ultimately only be defined by the woman experiencing it. Birth trauma leads to negative mental health and wellbeing outcomes for women, their children and their partners. Impacts on maternal mental health and wellbeing include post-traumatic stress disorder (PTSD), post-partum depression (PPD) and bonding issues but also potential for post-traumatic growth (PTG). Traumatic births can have a negative impact on breastfeeding experiences due to poorer maternal physical and psychological wellbeing. If breastfeeding does not go as planned, it can be associated with a sense of failure, guilt or shame. Therefore, infant feeding experiences may have the potential to either heal or compound the impact of traumatic births on maternal mental health and well-being.

**Methods:**

The guidelines for scoping reviews from the JBI Manual for Evidence Synthesis will be followed. A key word search strategy will be used to search: Academic Search Complete; APA PsychInfo; MEDLINE; CINAHL and APA PsychArticles databases; Google scholar, Bielefeld Academic Search Engine (BASE) and the National Institute for Health and Care Excellence (NICE) website. English language and date restrictions (2000-current) will be used. Studies will be screened in Covidence by two independent reviewers. Titles and abstracts and full-text articles will be independently screened by two reviewers. Data will be extracted and presented graphically using figures and tables. Narrative summary text will accompany the tables and figures.

**Conclusion:**

Given the potential for infant feeding experiences to either heal or compound the impact psychological birth trauma on maternal mental health and wellbeing, it is important to understand women’s experiences of these three constructs and how they interact with one another.

## Introduction

Childbirth can be a traumatic experience for some women ( Shorey & Wong, 2022). A traumatic birth experience can include objective birth experiences such as operative birth ( Chan et al., 2020), preterm birth ( Dikmen-Yildiz et al., 2018), or childbirth complications ( Chan et al., 2020). They also include the trauma of perinatal loss, including miscarriage and stillbirths ( Berry, 2022). Given the subjective nature of the birth experience, a traumatic birth can ultimately only be defined by the woman experiencing it ( Beck, 2004; Sun et al., 2023). A concept analysis based on 44 studies published between 2002–2022 defined the concept of psychological birth trauma as including the following attributes: (1) Women’s subjective feelings; (2) Intertwined painful emotional experiences; (3) Originate in the birth process; (4) Last until postpartum. Within this conceptualisation, psychological birth trauma emphasises women’s subjective experiences above the objective aspects of the birth process. The same events that occur during birth are perceived differently by every woman and childbirth that appears normal and straightforward to healthcare professionals can be perceived as traumatic by the woman ( Sun et al., 2023).

Psychological birth trauma can lead to negative mental health and relationship outcomes for women, their children and their partners ( Alcorn et al., 2010; Ayers, 2017; de Graaff et al., 2018; Leinweber et al., 2022; Shorey & Wong, 2022). The experience of a traumatic birth is associated with difficult emotions for women such as feeling like a “bad” mother, struggling to bond with their baby as well as postpartum anxiety, postpartum depression (PPD), and post-traumatic stress disorder (PTSD) ( Ayers et al., 2016; Frankham et al., 2023; Leach et al., 2017). These experiences bring added challenges to what is already a vulnerable transitional time for women ( Huschke et al., 2020; Molloy et al., 2021; World Health Organization, 2022). The cost of untreated maternal mental illness impacts both families and society as a whole ( Make Mothers Matter, 2023). It therefore represents a significant public health problem. International prevalence rates for PTSD are between 3 and 4.7% ( Grekin & O’Hara, 2014; Heyne et al., 2022; Yildiz et al., 2017). Despite the prevalence and impact of maternal mental health issues, most women globally do not receive the care and support they need ( World Health Organization, 2022) and perinatal mental health conditions are still under recognised and undertreated ( Wilson et al., 2024).

While birth trauma is associated with adverse outcomes, there is also potential for post-traumatic growth (PTG) to occur following psychological birth trauma. This is a positive psychological change that can occur alongside the distress associated with challenging life circumstances ( Beck et al., 2018; Beck & Watson, 2016; Tedeschi & Calhoun, 2004). PTG consists of five dimensions: appreciation of life, relating to others, personal strength, new possibilities, and spiritual change ( Tedeschi & Calhoun, 2004). PTG can occur following psychological birth trauma and can lead to increased self-confidence and pride, better relationship with partners, friends, children, and stronger faith and a better reported understanding of spiritual and religious matters ( Beck & Watson, 2016; Ketley et al., 2024). At a societal level, giving voice to birth stories that include both trauma and PTG may contribute to the development of varied and positive narratives around birth ( Ketley et al., 2024).

Traumatic births can have a negative impact on postpartum infant feeding experiences, including breastfeeding, Commercial Milk Formula feeding or combination feeding experiences due to poorer maternal physical and psychological wellbeing ( Norman et al., 2022, 2025). Both postnatal PTSD and PPD are associated with lower breastfeeding initiation and duration rates ( Cook et al., 2018; Garthus-Niegel et al., 2018). If breastfeeding does not go as planned, it can be associated with challenges, including breastfeeding grief ( Aderibigbe, 2026), a sense of failure, guilt or shame ( Jackson et al., 2021; Leeming et al., 2022). However, breastfeeding can also contribute to physical and psychological benefits for both mothers and babies, such as improved mother-infant bonding, increased maternal confidence, and reduced risk of infection, cancer and diabetes ( Del Ciampo & Del Ciampo, 2018; Scarborough et al., 2022). Therefore, it is also important to consider the role infant feeding experiences might play in fostering PTG following psychological birth trauma.

To date, there has been limited research exploring the interactions between infant feeding experiences and maternal mental health and wellbeing outcomes in women who experience psychological birth trauma. Infant feeding experiences have the potential to either heal or compound the impact psychological birth trauma can have on maternal mental health and wellbeing outcomes. Therefore, it is important to understand women’s experiences of these three constructs and how they interact with one another in order to support infant feeding and maternal mental health and wellbeing in women with psychological birth trauma.

### Objective

This scoping review will explore the breadth and nature of the experiences of and interactions between infant feeding experiences, maternal mental health and well-being in women who experience psychological birth trauma. It will seek to understand women’s experiences of infant feeding and maternal mental health and wellbeing following psychological birth trauma, identify proposed mechanisms and conceptual pathways, and highlight gaps for future research.

### Research questions

The overarching research question for this review is: What is the current evidence explaining the interactions between infant feeding experiences and maternal mental health and well-being outcomes in women who experience psychological birth trauma? We will seek to understand and gain insight into: a) what experiences support maternal wellbeing (including PTG) following psychological birth trauma? And b) how have women’s narratives related to their experiences infant feeding and mental health and wellbeing following psychological birth trauma been shared and presented within the literature?

## Methods and analysis

### Design

This review will follow Joanna Briggs Institute (JBI) guidelines for scoping reviews ( Peters et al., 2020). It will be reported in accordance with the Preferred Reporting Items for Systematic Reviews (PRISMA) extension for scoping reviews, PRISMA-ScR ( Tricco et al., 2018).

### Inclusion criteria

Any study examining or exploring interactions between traumatic birth, infant feeding and maternal mental health and well-being outcomes. Studies will only be included if the participants have reported experience of birth trauma. Reviews published in English since 2000 will be considered for inclusion. This date restriction was chosen based on the dates of studies identified in a recent conceptual analysis on psychological birth trauma ( Sun et al., 2023).

**Participants**

Any birthing person who has experienced a traumatic birth.

**Phenomena of interest**

This review is focused on: psychological birth trauma (women’s subjective feelings of having a traumatic birth experience with intertwined painful emotional experiences that originate in the birth process and last until postpartum), infant feeding experiences (including breastfeeding, bottle feeding or combination feeding) and maternal mental health (diagnosed or self-reported mental health symptoms including PTSD, Postnatal Depression and anxiety) and well-being (self-reported or measured mental wellbeing including post traumatic growth) outcomes.

**Outcomes**

Infant feeding experiences and maternal mental health and well-being outcomes in women with experience of psychological birth trauma.

**Context/Setting**

Studies conducted in any setting or context will be eligible for inclusion.

**Types of studies**

Any peer reviewed study, including qualitative, quantitative or mixed methods, examining or exploring the interaction or relationship between traumatic birth, infant feeding and maternal mental health and well-being outcomes. Identified reviews, including systematic review, scoping review or meta-analysis, will not be included but their reference lists will be searched, and relevant peer reviewed studies will be extracted and assessed for inclusion.

### Exclusion criteria

Studies that do not report on the interaction between women’s experiences of birth trauma, infant feeding and maternal mental health outcomes together. Studies not available in English.

### Search strategy

A three- phase search process will be used to locate both published and unpublished studies ( Aromataris et al., 2024). An initial search was carried out on Academic Search Complete; APA PsychInfo; MEDLINE; CINAHL and APA PsychArticles to identify articles on the topic. The initial keywords are informed by the relevant identified studies and by a recent concept analysis of psychological birth trauma ( Sun et al., 2023).

A full search strategy was then developed and reviewed by topic experts and knowledge users who are professionals in this area ( Tables 1 and 2).

**Table 1.** Developing the search strategy.

Search concepts	Birth trauma	Infant feeding	Maternal mental health outcomes
**Search terms**	“Trauma* birth” OR “birth trauma” OR “trauma* labour” OR “trauma labor” OR “labor trauma” OR “labour trauma” OR “Psychological birth trauma” OR “traumatic childbirth,” OR “traumatic delivery” OR “childbirth trauma” OR “delivery trauma”	Breastfeeding OR Breast-feeding OR Breastfeed OR Breast-feed OR Breastfed OR Breast-fed OR Lactation OR “Infant feed*” OR Infant-fed OR “Breastfeeding support” OR “Formula Fed” OR “Formula Feeding” OR “Commercial Milk Formula” OR “Bottle fed” OR “Bottle feeding”	“Post-traumatic Stress Disorder” OR “Postnatal Post-traumatic Stress Disorder” OR PTSD OR “Postnatal PTSD” OR Depression OR “Postnatal Depression” OR “Postpartum Depression” OR “Post-partum Depression” OR “Post partum Depression” OR “Post-natal Depression” OR PPD OR “Depressive Symptoms” OR Symptoms of depression” OR “Anxiety” OR “Anxiety Symptoms” OR “Symptoms of anxiety” OR “Anxiety Disorder” OR Wellbeing OR “mental health” OR “Post-Traumatic Growth” OR “posttraumatic growth” OR PTG

**Table 2.** Search strategy.

Search	Query
**#1**	**Title/Abstract:** “Trauma* birth” OR “birth trauma” OR “trauma* labour” OR “trauma labor” OR “labor trauma” OR “labour trauma” OR “Psychological birth trauma” OR “traumatic childbirth,” OR “traumatic delivery” OR “childbirth trauma” OR “delivery trauma”
**#2**	**Title/Abstract:** Breastfeeding OR Breast-feeding OR Breastfeed OR Breast-feed OR Breastfed OR Breast-fed OR Lactation OR “Infant feed*” OR Infant-fed OR “Breastfeeding support” OR “Formula Fed” OR “Formula Feeding” OR “Commercial Milk Formula” OR “Bottle fed” OR “Bottle feeding”
**#3**	“Post-traumatic Stress Disorder” OR “Postnatal Post-traumatic Stress Disorder” OR PTSD OR “Postnatal PTSD” OR Depression OR “Postnatal Depression” OR “Postpartum Depression” OR “Post-partum Depression” OR “Post partum Depression” OR “Post-natal Depression” OR PPD OR “Depressive Symptoms” OR Symptoms of depression” OR “Anxiety” OR “Anxiety Symptoms” OR “Symptoms of anxiety” OR “Anxiety Disorder” OR Wellbeing OR “mental health” OR “Post-Traumatic Growth” OR “posttraumatic growth” OR PTG
**#4**	#1 AND #2 AND #3
**Limits**	2000-present

Database-specific searches will be conducted for Academic Search Complete; APA PsychInfo; MEDLINE; CINAHL and APA PsychArticles. A grey literature search will be undertaken on Google scholar, BASE and the National Institute for Health and Care Excellence (NICE) website. The reference list of all included reviews will be searched manually for studies that meet inclusion criteria. Date restrictions (2000-current) will be used. One full search has been included in this protocol ( Table 3).

**Table 3.** Full sample search string for APA PsychInfo.

Search	Query	Records retrieved
**#1**	**Title/Abstract:** “Trauma* birth” OR “birth trauma” OR “trauma* labour” OR “trauma labor” OR “labor trauma” OR “labour trauma” OR “Psychological birth trauma” OR “traumatic childbirth,” OR “traumatic delivery” OR “childbirth trauma” OR “delivery trauma”	**574**
**#2**	**Title/Abstract:** Breastfeeding OR Breast-feeding OR Breastfeed OR Breast-feed OR Breastfed OR Breast-fed OR Lactation OR “Infant feed*” OR Infant-fed OR “Breastfeeding support” OR “Formula Fed” OR “Formula Feeding” OR “Commercial Milk Formula” OR “Bottle fed” OR “Bottle feeding”	**10,039**
**#3**	**Title/Abstract:** “Post-traumatic Stress Disorder” OR “Postnatal Post-traumatic Stress Disorder” OR PTSD OR “Postnatal PTSD” OR Depression OR “Postnatal Depression” OR “Postpartum Depression” OR “Post-partum Depression” OR “Post partum Depression” OR “Post-natal Depression” OR PPD OR “Depressive Symptoms” OR Symptoms of depression” OR “Anxiety” OR “Anxiety Symptoms” OR “Symptoms of anxiety” OR “Anxiety Disorder” OR Wellbeing OR “mental health” OR “Post-Traumatic Growth” OR “posttraumatic growth” OR PTG	**379,109**
**#4**	#1 AND #2 AND #3	**15**
**Limits**	2000-present	

### Screening

All identified articles will be uploaded to Covidence. Duplicates will be removed. Title and abstracts of the remaining articles will be screened by two independent reviewers. The full text of all articles that meet the inclusion criteria will then be reviewed and screened by two independent reviewers on Covidence. If there are any disagreements between reviewers during the screening process, a third reviewer will assess the relevant records, and consensus will be reached on eligibility through discussion. Reasons for article exclusion will be reported. Results of the screening process will be presented in a PRISMA flow diagram ( Tricco et al., 2018).

### Data extraction

Data extraction will be conducted by two independent reviewers using a data extraction template that will be adapted by reviewers based on the standardised JBI data extraction tools, the review objectives and research questions ( Table 4). The data extraction tool will be piloted and revised before use and during the process of data extraction, if needed. If disagreements arise between reviewers during data extraction, they will be resolved through discussion or with a third reviewer. If necessary, authors of papers will be contacted to request missing or additional information.

**Table 4.** Data extraction instrument.

Author/Year	Objectives	Participants (characteristics/total number)	Setting/context	Study appraisal rating	Definition of traumatic birth	Definition and measurement of infant feeding experience	Definition and measurement of mental health outcome	Results: Experiences of and Interactions between traumatic birth, infant feeding and maternal mental health outcome(s)	Women’s narratives around experiences a birth trauma, infant feeding and maternal mental health and wellbeing

### Quality assessment

Given that this scoping review aims to map the nature and extent of available evidence in relation to the interaction between birth trauma, infant feeding experiences and maternal mental health and wellbeing and will not be assessing intervention effectiveness or strength of associations, a quality assessment will not be necessary ( Peters et al., 2020).

### Data analysis

The information captured by the data extraction tool will be mapped and summarised according to the aims and objectives of this review. The included studies will be summarised based on the studies’ characteristics, including the year, study aim and objectives, participants, and context.

Narrative summaries will outline the identified interactions between traumatic birth experiences, infant feeding experiences and maternal mental health outcomes. This information will be analysed to identify key themes and findings across the included studies. Where suitable, data will be presented graphically using figures and tables. Narrative summaries will explain the results presented in all figures and tables.

A reflexive approach will be adopted throughout the review process. The reviewers will critically reflect on the selection of studies, interpretation of the literature and the potential biases inherent in the synthesis process. Reflexivity will involve the reviewers’ being transparent about their perspectives, decisions made during the review process, and how these may influence findings. A reflexive statement will be included in the review discussing these considerations to provide a richer understanding of the choices made and how these may shape the conclusions drawn.

## Data summary

A narrative synthesis will be presented to summarise the relationships and interactions between infant feeding experiences and maternal mental health outcomes in women who have experienced psychological birth trauma. The results of the review will inform our understanding of women’s experience of infant feeding and mental health and wellbeing following a traumatic birth, future research and our understanding of how the interactions between infant feeding experiences and maternal mental and wellbeing health can be harnessed to improve outcomes for women who experience psychological birth trauma.

## Data Availability

This is a scoping review protocol and does not have any associated data.
