# Supplementary material for: Understanding the Interaction Between Infant Feeding and Maternal Mental Health and Well-Being in Women who Experience Psychological Birth Trauma: A Scoping Review Protocol
Source: HRB Open Res. 2026 Apr 21;9:39. [Version 1] doi: 10.12688/hrbopenres.14414.1 (PMC13247567; doi:10.12688/hrbopenres.14414.1)
Supplement: Peer review history

# Reviewer report: Reviewer response for version 1

**DOI:** 10.21956/hrbopenres.15878.r55493

**Published:** 2026-06-08

**Authors:** Susanna Foxworthy Scott

**Affiliations:** 1 Butler University, Indianapolis, Indiana, USA

This is a well-developed protocol addressing an important and underexplored relationship between infant feeding and traumatic birth. The section on post-traumatic growth (PTG) was a particular strength.

 One area that could further enrich the background is the relationship between traumatic birth and physiological low milk production, specifically, the disruption of lactogenesis II that can follow complicated or traumatic deliveries. Acknowledging this mechanistic pathway alongside the psychological dimensions would strengthen the theoretical grounding of the review. The authors may also wish to consider whether including search terminology related to delayed lactogenesis and intrapartum factors affecting milk onset would meaningfully expand the yield of included studies.

Is the study design appropriate for the research question?

Yes

Is the rationale for, and objectives of, the study clearly described?

Yes

Are sufficient details of the methods provided to allow replication by others?

Yes

Are the datasets clearly presented in a useable and accessible format?

Yes

Reviewer Expertise:

health communication, low milk supply, lactation, birth narratives

I confirm that I have read this submission and believe that I have an appropriate level of expertise to confirm that it is of an acceptable scientific standard.

# Reviewer report: Reviewer response for version 1

**DOI:** 10.21956/hrbopenres.15878.r55222

**Published:** 2026-06-03

**Authors:** Sarah Myers

**Affiliations:** 1 Max Planck Institute for Evolutionary Anthropology, Leipzig, Germany

I think this is an interesting and important topic. I offer some thoughts which the authors may wish to consider when approaching the scoping review and I hope they find useful, but broadly speaking the methodology appears sound and the outcome will be a useful step in the literature. 

 While I find the need for such a scoping review compelling, I do find the framing somewhat limited. The authors state that their goal is to “understand women’s experiences of these three constructs”, the constructs being infant feeding, psychological birth trauma, and mental health; however, positive infant feeding experience/outcomes appear to primarily framed in terms of breastfeeding. To lead with an assumption of breastfeeding being the default intention of birthing individuals and that birth trauma will only meaningfully disrupt the experience of breastfeeding, is to ignore the experiences of those who did not ever wish to breastfeed and those whose decision to switch to formula/combined feeding is not experienced as negative per se, but rather their subsequent experience of formula is (which is common in the absence of birth trauma: Myers, S., Page, A.E. and Emmott, E.H., 2021. The differential role of practical and emotional support in infant feeding experience in the UK. Philosophical Transactions of the Royal Society B: Biological Sciences, 376(1827)). I also see the potential to misattribute the cessation of breastfeeding to trauma, if it is assumed that it would otherwise have continued, when in fact at a country level it is not uncommon for the majority of infants to not exclusively breastfeed for the WHO recommended period. Moreover, while I do not doubt that there are benefits associated with breastfeeding, there are also costs and it is not always what is best for the birthing parent – I would also note that Del Ciampo and Del Ciampo (2018) is not a confidence inspiring citation, having no methodology and a clear agenda. I appreciate that the authors state they will take a reflexive approach and this is to be encouraged, particularly when it is the lived experiences of those suffering birth trauma that should be foregrounded; however, it would have been nice to see a little more of this in the opening set up too.

 I would caution against implying causality when it comes to the correlations found between the three constructs in quantitative studies. The vast majority of quantitative research in this area has not been conducted in a manner the permits causal inference and much of it is reported in such a way as to make any reported correlations/associations uninterpretable – if an identified paper does not employ methodology incorporating causal modelling/directed acyclic graphs/potential outcomes then its results should be treated with extreme caution (see: Tennant, P.W., Murray, E.J., Arnold, K.F., Berrie, L., Fox, M.P., Gadd, S.C., Harrison, W.J., Keeble, C., Ranker, L.R., Textor, J. and Tomova, G.D., 2021. Use of directed acyclic graphs (DAGs) to identify confounders in applied health research: review and recommendations. International journal of epidemiology, 50(2), pp.620-632)). 

 Following on from this point, I would be inclined to disagree that quality assessment is not required; while exclusion on the basis of perceived quality is perhaps not warranted (particularly given the limited number of articles covering all three concepts), quality is important when considering evidence availability. A quantitative paper reporting confounded statistical modelling is potentially indicative of limited to no evidence (though it may contain useful data if open access); to consider such a paper as reporting evidence of equal value to a statistically robust paper or a nuanced qualitative study is at best to overstate the available evidence, and at worst to embed misguided interpretations into the literature.

 Finally, please note I responded ‘partly’ to the question ‘Are sufficient details of the methods provided to allow replication by others?’ simply because elements of the methodology, e.g. data extraction, can only be fully described in the final study’s presentation – it is not a criticism.

Is the study design appropriate for the research question?

Yes

Is the rationale for, and objectives of, the study clearly described?

Yes

Are sufficient details of the methods provided to allow replication by others?

Partly

Are the datasets clearly presented in a useable and accessible format?

Not applicable

Reviewer Expertise:

Infant feeding, postnatal depression, social support, quantitative methods, causal inference.

I confirm that I have read this submission and believe that I have an appropriate level of expertise to confirm that it is of an acceptable scientific standard.
